# Predictors of inotrope use in patients undergoing concomitant coronary artery bypass graft (CABG) and aortic valve replacement (AVR) surgeries at separation from cardiopulmonary bypass (CPB)

**DOI:** 10.1186/1749-8090-4-24

**Published:** 2009-06-12

**Authors:** Imdad Ahmed, Chad M House, William B Nelson

**Affiliations:** 1 Department of Hospital Medicine, Regions Hospital, University of Minnesota Medical School, St Paul, Minnesota, USA; 2 Department of Cardiology, Regions Hospital, St Paul, Minnesota, USA; 3 Department of Cardiology, Regions Hospital, University of Minnesota Medical School, Minneapolis, Minnesota, USA

## Abstract

**Background:**

Left ventricular dysfunction is common after coronary artery bypass graft and valve replacement surgeries and is often treated with inotropic drugs to maintain adequate hemodynamic status. In this study, we aimed to identify the demographic, clinical, laboratory, echocardiographic and hemodynamic factors that are associated with use of inotropic drugs in patients undergoing concomitant coronary artery bypass graft and aortic valve replacement surgery.

**Methods:**

The study included 97 patients who had undergone concomitant coronary artery bypass graft and aortic valve replacement at Regions Hospital, University of Minnesota Medical School from January 2006 to December 2008. All data were collected retrospectively after reviewing electronic medical records. Inotropic support was defined as the use of dopamine [greater than or equal to] 5 ug/kg/min; any dose of epinephrine, norepinephrine, dobutamine, and milrinone at the separation from cardiopulmonary bypass.

**Results:**

Inotropic support was used in a total of 50 patients (52%) at the separation from cardiopulmonary bypass. Average age of the patients requiring inotropic support was 72.2 +/- 8.8 years. The study identified four significant, independent predictors of inotrope use: (1) Cardiac index [less than or equal to]2.5 L/min/m2, (2) LVEDP [greater than or equal to] 20 mm Hg, (3) LVEF [less than or equal to]40%, and (4) CKD stage 3 to 5.

**Conclusion:**

We identified four independent risk factors for postoperative use of inotropic support in patients undergoing concomitant coronary artery bypass graft and arotic valve replacement surgery at the separation from cardiopulmonary bypass. The study results will be helpful to prospectively identify patients who will likely to require inotropic support at the separation from cardiopulmonary bypass.

## Background

The incidence of inotrope support is proportionally higher in patients undergoing combined coronary artery bypass graft (CABG) and valve surgery as compared to CABG alone[1]. Left ventricular dysfunction often occurs after cardiopulmonary bypass (CPB), requiring the use of inotropic drugs to achieve adequate hemodynamic status [2]. In contrast to patients with chronic ischemic heart disease, left ventricular dysfunction in patients with moderate-to-severe valvular heart disease does not improve immediately after surgery [1,3]. In patients with normal preoperative ventricular function, contractile dysfunction can occur between 4–6 hours after surgery, and usually resolves around 24 hours postoperatively [4]. Although many clinicians choose to treat patients prophylactically with inotropic drugs[5], these drugs should be administered selectively as use of these drugs subjects the patients to an increased risk of tachycardia, dysarhythmia, and myocardial ischemia[6,7].

Older age, reduced ejection fraction, female gender, cardiomegaly, history of congestive heart failure, emergency operation, recent myocardial infarction, prolonged duration of CPB or aortic clamping, and left main coronary artery disease were found to be risk factors associated with need for inotropic support[2,8,9]. Pathophysiologic changes associated with moderate-to-severe valvular heart disease are longstanding and risk factors for inotropic support are likely to be different in patients undergoing concomitant CABG and valve replacement. A randomized, double-blinded study, by Butterworth et al., showed that the specific valve disease did not influence use of inotropic support after heart valve surgery [2].

The purpose of our study was to identify the demographic, clinical, laboratory, echocardiographic and hemodynamic factors that are associated with the use of inotropic support during separation from CPB in patients undergoing concomitant coronary artery bypass grafting (CABG) and aortic valve replacement (AVR) surgery.

## Methods

After institutional review board approval, detailed clinical, laboratory, echocardiographic and hemodynamic data were retrospectively examined. All patients had transthoracic echocardiography (TTE), right and left heart catheterizations and coronary angiogram within one month prior to the surgical procedure. A comprehensive transesophageal echocardiography (TEE) was performed in all patients before cardiopulmonary bypass.

Patients who had concomitant coronary artery bypass graft surgery (CABG) and aortic valve replacement (AVR), without requiring preoperative inotropic support or an intra-aortic balloon pump (IABP) were included. The study includes 97 patients (54 men and 43 women) who underwent concomitant CABG and AVR surgery from January, 2006 to December 2008 at the Regions Hospital of University Minnesota Medical School.

Electronic medical records of all patients were reviewed. Age, sex, body mass index (BMI), preoperative medication use and New York Heart Association (NYHA) class prior to the surgical procedure were collected. Hemoglobin and creatinine level prior to surgical procedure were recorded. A decrease in hemoglobin was calculated by recording the second hemoglobin level collected routinely within 6 hours after the surgical procedure. Chronic kidney disease (CKD) was assessed by glomerular filtration rate (GFR), as recommended by National Health and Nutrition Examination Survey (NHANES) [10]. We included chronic kidney disease stage 3 to stage 5 with a GFR less than 60 mL/min per 1.73 m^2 ^in the study for analysis. GFR was calculated by using Modification of Diet in Renal Disease study equation (MDRD) [11].

Transthoracic echocardiography (TTE) results were reviewed and the following data were collected: left ventricular ejection fraction (LVEF), wall motion abnormalities, moderate-to-severe mitral regurgitation (MR), left ventricular hypertrophy (LVH) and presence of pulmonary hypertension. Patients with hypokinesia, akinesia or dyskinesia in the echocardiographic (TTE) report were included as having wall motion abnormalities. Mitral regurgitation was reported as mild, moderate or severe. Only patients with moderate-to- severe MR were included in the study for analysis.

Results of the right and left heart catheterization were reviewed and following data were collected: left ventricular end diastolic pressure (LVEDP) and cardiac index (CI).

All surgical procedures were conducted under general endotracheal anesthesia with midline sternotomy approach. Full heparinization was carried out by an anesthesiologist, with a goal of activated clotting time of over 400. The Patient was placed in cardiopulmonary bypass and retrograde cardioplegia was delivered. Cold ice slush was used for topic myocardial protection. Patient was systemically drifted to 31–33 degree celsius throughout the cross- clamp time. Aortic valve replacement and coronary artery bypass graft were performed with standard techniques. After completion of AVR and CABG, aortic cross clamp was released. Temporary atrial and ventricular pacing wires were placed in a standard fashion. After appropriate rest period and gradual rewarming, the patient was weaned from cardiopulmonary bypass. Protamine was given to reverse the heparin effect and the patient was decannulated. After adequate hemostasis was achieved, the patient was transferred to the surgical intensive care unit.

Inotropic support was defined as the use of dopamine ≥ 5 μg/kg/min; any dose of epinephrine, norepinephrine, dobutamine, or milrinone. None of the patients received IABP support during the separation from CPB.

All patients were followed-up until March 31, 2009 and mortality data and cause of death were collected.

All statistical analyses were performed with Fischer's exact test and a logistic regression analysis. A *p *value < 0.05 was considered significant. Statistical analyses were conducted using STATA 10.0 software (STATA Statistics/Data analysis, Texas, USA)

## Results

Ninety-seven patients underwent concomitant CABG and AVR between January 2006 and December 2008. The demographics of this patient group are displayed in Table 1. The percentage of men (58%) was higher than women (42%) and the average age was 71.8 ± 10.1 years. Forty seven percent of patients had diabetes mellitus and 70% had hypertension. Pre-operative NYHA class assessment showed 11% patients were in class II, 69% in class III and 20% were in class IV. Seventy four (76%) patients had aortic stenosis, 16 (17%) patients had aortic regurgitation and 7 (7%) patients had both aortic stenosis and regurgitation.

**Table 1:** Patient Demographics

Age in years (± SD)	71.8 ± 10.1
Sex	

Male	56 (58%)

Female	41 (42%)

Diabetes Mellitus	46 (47%)

Hypertension	68 (70%)

NYHA class	

II	11 (11%)

III	67 (69%)

IV	19 (20%)

Aortic stenosis (AS)	74 (76%)

Aortic regurgitation (AR)	16 (17%)

Both AS and AR	7(7%)

Of 97 patients, 50(52%) patients required inotropic support at the separation of CPB. Women had slightly higher need of inotropic support than men (27% vs 25%). The average age of the patients requiring inotropic support was 72.2 ± 8.8 years.

Univariate analysis showed that patients ≥ 70 years old were more likely to get inotropic support (p = 0.02). Obese patients with BMI ≥ 30 Kg/m^2 ^and patients with CKD stage 3 to 5 were associated with increased use of inotropic support (p = 0.04 and p = 0.02 respectively). Analysis of the preoperative echocardiographic (TTE) data showed that patients with left ventricular ejection fraction (LVEF) ≤ 40% were more likely to receive inotropic support (p = 0.001). Presence of pulmonary hypertension (p = 0.69), moderate-to-severe mitral regurgitation (MR) (p = 0.37), left ventricular hypertrophy (LVH) (p = 0.11) and wall motion abnormalities (p = 0.86) were not associated with an increased use of inotropic support. Neither sex (p = 0.43) nor a preoperative diagnosis of diabetes (p = 0.61) appeared to be associated with the use of inotropic support. All patients had their hemoglobin level checked within 6 hours after the surgical procedure. A postoperative drop of hemoglobin ≥ 6 gm/dl compared to preoperative value, was found to have higher use of inotropic support (p = 0.02). Preoperative use of beta-blockade was not associated with an increased use of inotropic support (p = 0.23).

Analysis of hemodynamic data showed that patients with preoperative left ventricular end diastolic pressure (LVEDP) ≥ 20 mm Hg and cardiac index (CI) ≤ 2.5 liters per minute squared body surface area(L/min/m^2^) were found to be highly associated with increased use of inotropic support (p = 0.006 and 0.001 respectively). Univariate predictors of inotrope use are displayed in Table 2.

**Table 2:** Univariate Predictors of Inotrope Use

	Inotropic support group(n = 50)	Noninotropic support group(n = 47)	*P *value
Age (≥ 70 years)	31 (32%)	18 (19%)	0.02
			
Sex			
Male	24(25%)	19(20%)	0.43
Women	26(27%)	28(29%)	
			
BMI ≥ 30	28(29%)	14(14%)	0.04
			
Diabetes Mellitus	21(22%)	25(26%)	0.61
			
CKD stage 3–5 (GFR<60 mL/min/1.73 m^2^)	14(14%)	5(5%)	0.02
			
Pulmonary hypertension	18(19%)	15(15%)	0.69
			
LVEF ≤ 40%	45(46%)	31(32%)	0.001
			
LVH	36(37%)	43(44%)	0.11
			
Moderate to severe MR	21(22%)	15(15%)	0.37
			
Wall motion abnormalities	16(16%)	14(14%)	0.86
			
Drop of hemoglobin ≥ 6 gm/dl	29(30%)	16(16%)	0.02
			
Pre-operative use of beta blocker	26(27%)	31(32%)	0.23
			
LVEDP ≥ 20 mm Hg	45(46%)	31(32%)	0.006
			
CI ≤ 2.5 L/min/m2	29(30%)	15(15%)	0.001

Abbreviations: BMI, body mass index; CKD, chronic kidney disease; MR, mitral regurgitation; LVEF, left ventricular ejection fraction; LVH, left ventricular hypertrophy; LVEDP, left ventricular end diastolic pressure; CI, cardiac index

Stepwise multivariate logistic regression analysis identified 4 significant, independent predictors of inotrope use at separation from CPB (Table 3). Cardiac index ≤ 2.5 L/min/m2 (odds ratio: 3.1, 95% confidence interval {1.13–8.4}), LVEDP ≥ 20 mm Hg (3.58{1.16–9.03}), LVEF ≤ 40% (2.76{1.11–6.86}), and CKD stage 3–5 (3.26{1.07–9.95}) were all associated with inotropic support necessity in patients who had undergone concomitant CABG and AVR at the separation of CPB.

**Table 3:** Multivariate Predictors of Inotrope Use

Variable	Odds Ratio(95% Confidence Interval)	*P *Value
Cardiac index ≤ 2.5 L/min/m2	3.1(1.13–8.4)	0.03
LVEDP ≥ 20 mm Hg	3.58(1.16–9.03)	0.02
LVEF ≤ 40%	2.76(1.11–6.86)	0.01
CKD stage 3–5(GFR<60 mL/min/1.73 m^2^	3.26(1.07–9.95)	0.04

Postoperatively, out of 97 patients, 16 patients (16.5%) developed paroxysmal atrial fibrillation (11 with inotropic support and 5 without inotropic support), 7 patients (7%) developed non-sustained ventricular tachycardia (5 with inotropic support and 2 without iniotropic support), 4 patients (4%) developed acute renal failure (all with inotropic support) and 3(3%) patients were diagnosed with gram negative pneumonia (2 with inotropic support and one without inotropic support). Three patients (3%) developed postoperative mediastinal bleeding (2 with inotropic support and one without inotropic support) and all required reoperation to control bleeding. Six patients (6%) developed postoperative sick sinus syndrome (3 with inotropic support and 3 without inotropic support) and required permanent pacemaker placement. Eight patients (8%) developed postoperative acute decompensated heart failure and required aggressive intravenous diuresis (all with inotropic support).

Table 4 shows the causes of death of all patients, days from the surgical procedure and whether or not they required inotropic support after the surgery. Out of 97 patients who underwent concomitant CABG and AVR, 10 patients died after the surgical procedures. One patient died within 24 hours after the surgical procedure from congestive heart failure and required inotropic support after the surgery. One patient died of congestive heart failure on the second postoperative day and required inotropic support after the surgery. Out of total ten deaths, 8 patients died after the hospital discharge.

**Table 4:** Causes of death of all patients undergoing concomitant CABG and AVR

Inotropic support	Days from the surgery	Cause of death
Yes	1	Congestive heart failure
Yes	3	Congestive heart failure
No	183	Congestive heart failure
No	227	Colon cancer
Yes	281	Septicemia
Yes	347	Congestive heart failure
No	546	Esophageal cancer
No	581	Septicemia
No	738	Congestive heart failure
No	756	Gastrointestinal bleeding

## Discussion

In a retrospective study of 97 patients undergoing concomitant CABG and AVR, 52% received inotropic support. We have identified 4 independent preoperative risk factors for the use of inotropic support at the separation from CPB. Cardiac index ≤ 2.5 L/min/m2, LVEDP ≥ 20 mm Hg, LVEF ≤ 40% and CKD stage 3 to 5 were all associated with an increased use of inotropes. The results of the study will help cardiothoracic surgeons, anesthesiologists and intensivists to prospectively identify those patients who will likely to require inotropic support at the separation from CPB. Compared to previous published reports, the availability of hemodynamic data is the strength of the study. Coronary angiogram, right and left heart catheterizations are routinely performed in patients undergoing concomitant CABG and AVR and the additional hemodynamic data will be very useful in identifying those high risk patients. In a study of 1,009 patients conducted by McKinlay et al., it was found that incidence of inotrope use was greater in patients undergoing combined CABG and valve surgery as compared to CABG alone and combined CABG and valve surgery had longer cross-clamp time [1]. We did not include cross-clamp time and duration of CPB in our analysis as these data were not consistently found in our electronic medical records. In patients with moderate-to-severe aortic stenosis, left ventricular hypertrophy and reduced left ventricular compliance do not improve immediately after valve replacement due to persistent high afterload. Similarly, in patients with aortic valve insufficiency, left ventricular dilatation persists even after aortic valve replacement [2]. In patients with longstanding coronary artery disease and moderate-to-severe valvular heart disease, left ventricular dysfunction likely will be greater as compared to either alone.

Left ventricular dysfunction, as evidenced by reduced ejection fraction and low cardiac index, was found to be an independent predictor of inotropic support in our study. Left ventricular dysfunction was found to be a powerful predictor of inotrope use in earlier studies in isolated CABG or valve replacement surgery [8]. LVEF<35% was found to be an independent predictor of inotropic support in patients undergoing CABG in the study conducted by McKinlay et al [1]. Rao et al. studied a cohort of 4558 consecutive patients undergoing isolated CABG surgery and found several risk factors for increased use of inotropic support, including left ventricular ejection fraction <20%, age >70 years and female gender Although ventricular dysfunction after CPB is largely a transient phenomenon related to ischemia, left ventricular dilatation and reduced compliance, poor LV function continues to be one of the most significant predictors of post-operative inotropic support [9].

Advancing age was found to be a significant risk factor of inotrope use in the previous studies [2,8,9] and our study showed the similar result. In the univariate analysis, age ≥ 70 years was significantly associated with inotrope use, which was similar to the result found in the study conducted by Rao et al [9]. We did not find any significant association between particular gender and use of inotrope as compared to increased risk in female gender in the study by Rao et al [9].

Elevated LVEDP was shown to be an independent predictor of mortality after cardiac surgery independently of LVEF [12]. One frequent cause of elevated LVEDP is left ventricular hypertrophy (LVH), which is a risk factor for diastolic dysfunction [13]. LVH was present in 81% of our study patients. We did not include diastolic dysfunction as a risk factor in our study as it was not consistently mentioned as "normal" or "abnormal" in the echocardiographic (TTE and TEE) reports. LVEDP could be associated with systolic dysfunction, but patients with normal systolic function and elevated LVEDP might be at higher risk of mortality after cardiac surgery because of the deleterious effect of elevated LVEDP with associated filling abnormalities [12]. These abnormal loading conditions may predispose the patient very sensitive to perioperative, often abrupt changes in loading conditions with hypovolaemia on one hand and volume overload on the other [12,14].

Obesity is well known to be major risk factor for cardiovascular disease and in our study we have shown obesity to be a predictor of postoperative inotrope use in the univariate analysis. In a retrospective cohort study, Pan et al. concluded that obesity in diabetic patients is an independent predictor of increased postoperative morbidity after CABG surgery [15]. CKD stage 3 to 5, with GFR < 60 mL/min/1.73 m^2 ^was an independent predictor of inotrope use in our study population. In a study published by Yeo et al. GFR was found to be a significant risk factor for operative mortality in patients undergoing CABG and CKD stages 3, 4, and 5 were progressively associated with increased operative mortality [16].

Out of total ten deaths in our study population, two patients died in the same hospital admission and both of these patients required inotropic support after the surgical procedures. Although, it might be possible that inotropic support is associated with higher postoperative mortality in patients undergoing concomitant CABG and AVR, a prospective study with larger number of patients will be required to better establish this hypothesis.

Our study, inevitably, has limitations. The study was retrospective and data were obtained from a single institution. It was not conducted in a controlled, prospective, randomized fashion. We have retrospectively reviewed the results of echocardiography and cardiac catheterization reports which were performed and read by different cardiologists. There is a possibility of observer bias. Intiation of inotrope might be at discretion of attending anesthesiologists or intensivists and thus, selection bias also cannot be overlooked.

In conclusion, low ejection fraction and left ventricular dysfunction are frequently observed after CPB. Immediate effects of CPB on LV systolic function are well known, but the effects on diastolic dysfunction are less clear [17].

We believe that results of our study may be used to prospectively identify patients undergoing concomitant CABG and AVR who will likely to require inotropic support at the separation of CPB.

## Competing interests

The authors declare that they have no competing interests.

## Authors' contributions

IA did the study design, data collection, statistical analysis, and manuscript writing. CMH and WBN reviewed the statistical analysis and the manuscript and made necessary corrections. All authors read and approved the final manuscript.

## References

[B1] McKinlay KH, Schinderle DB, Swaminathan M, Podgoreanu MV, Milano CA, Messier RH, El-Moalem H, Newman MF, Clements FM, Mathew JP (2004). Predictors of inotrope use during separation from cardiopulmonary bypass. J Cardiothorac Vasc Anesth.

[B2] Butterworth JF, Legault C, Royster RL, Hammon JW (1998). Factors that predict the use of positive inotropic drug support after cardiac valve surgery. Anesth Analg.

[B3] Braunwald E, Rutherford JD (1986). Reversible ischemic left ventricular dysfunction: Evidence for the "hibernating myocardium". J Am Coll Cardiol.

[B4] Royster RL (1993). Myocardial dysfunction following cardiopulmonary bypass: recovery patterns, predictors of inotropic need, theoretical concepts of inotropic administration. J Cardiothorac Vasc Anesth.

[B5] Hardy JF, Belsle S (1993). Inotropic support of the heart that fails to successfully wean from cardiopulmonary bypass:The Montreal Heart Institute experience. J Cardiothorac Vas Anesth.

[B6] Lazar HL, Buckberg GD, Foglia RP, Manganaro AJ, Maloney JV (1981). Detrimental effects of premature use of inotropic drugs to discontinue cardiopulmonary bypass. J Thorac Cardiovasc Surg.

[B7] Lewis KP (1993). Early intervention of inotropic support in facilitating weaning from cardiopulmonary bypass: The New England Deaconess Hospital experience. J Carthiothorac Vasc Anesth.

[B8] Royster RL, Butterworth JF, Prough DS, Johnston WE, Thomas JL, Hogan PE, Case LD, Gravlee GP (1991). Preoperative and intraoperative predictors of inotropic support and long-term outcome in patients having coronary artery bypass grafting. Anesth Analg.

[B9] Rao V, Ivanov J, Weisel RD, Ikonomidis JS, Christakis GT, David TE (1996). Predictors of low cardiac output syndrome after coronary artery bypass. J Thorac Cardiovasc Surg.

[B10] Coresh J, Astor BC, Greene T, Eknoyan G, Levey AS (2003). Prevalence of chronic kidney disease and decreased kidney function in the adult US population: Third National Health and Nutrition Examination Survey. Am J Kidney Dis.

[B11] Levey AS, Coresh J, Greene T, Stevens LA, Zhang YL, Hendriksen S, Kusek JW, Van Lente F (2006). Using standardized serum creatinine values in the modification of diet in renal disease study equation for estimating glomerular filtration rate. Ann Intern Med.

[B12] Salem R, Denault AY, Couture P, Belisle S, Fortier A, Guertin M-C, Carrier M, Martineau R (2006). Left ventricular end-diastolic pressure is a predictor of mortality in cardiac surgery independently of left ventricular ejection fraction. Br J Anes.

[B13] Chenzbraun A, Pinto FJ, Popylisen S, Schnittger I, Popp RL (1994). Filling patterns in left ventricular hypertrophy: a combined acoustic quantification and Doppler study. J Am Coll Cardiol.

[B14] Redfield MM, Jacobsen SJ, Burnett JC, Mahoney DW, Bailey KR, Rodeheffer RJ (2003). Burden of systolic and diastolic ventricular dysfunction in the community: appreciating the scope of the heart failure epidemic. JAMA.

[B15] Pan W, Hindler K, Lee VV, Vaughn WK, Collard CD (2006). Obesity in diabetic patients undergoing coronary artery bypass graft surgery is associated with increased postoperative morbidity. Anesthesiology.

[B16] Yeo KK, Li Z, Yeun JY, Amsterdam E (2008). Severity of chronic kidney disease as a risk factor for operative mortality in nonemergent patients in the California coronary artery bypass graft outcomes reporting program. Am J Cardiol.

[B17] Djaiani GN, McCreath BJ, Ti LK, Mackensen BG, Podgoreanu M, Phillips-Bute B, Mathew JP (2002). Mitral flow propagation velocity identifies patients with abnormal diastolic function during coronary artery bypass graft surgery. Anesth Analg.

